# BMP-2 Enhances Osteogenic Differentiation of Human Adipose-Derived and Dental Pulp Stem Cells in 2D and 3D *In Vitro* Models

**DOI:** 10.1155/2022/4910399

**Published:** 2022-03-04

**Authors:** Sara Martin-Iglesias, Lara Milian, María Sancho-Tello, Rubén Salvador-Clavell, José Javier Martín de Llano, Carmen Carda, Manuel Mata

**Affiliations:** ^1^BCMaterials, Parque Científico UPV/EHC, Bizkaia, Spain; ^2^Department of Pathology, Faculty of Medicine and Dentistry, Universitat de València, Valencia, Spain; ^3^INCLIVA, Instituto de Investigación Sanitaria de Valencia, Valencia, Spain; ^4^CIBER-BBN, Centro de Investigación Biomédica en Red en Bioingeniería, Biomateriales y Nanomedicina, ISCIII, Madrid, Spain; ^5^CIBERES, Centro de Investigación Biomédica en Red en Enfermedades Respiratorias, ISCIII, Madrid, Spain

## Abstract

Bone tissue provides support and protection to different organs and tissues. Aging and different diseases can cause a decrease in the rate of bone regeneration or incomplete healing; thus, tissue-engineered substitutes can be an acceptable alternative to traditional therapies. In the present work, we have developed an *in vitro* osteogenic differentiation model based on mesenchymal stem cells (MSCs), to first analyse the influence of the culture media and the origin of the cells on the efficiency of this process and secondly to extrapolate it to a 3D environment to evaluate its possible application in bone regeneration therapies. Two osteogenic culture media were used (one commercial from Stemcell Technologies and a second supplemented with dexamethasone, ascorbic acid, glycerol-2-phosphate, and BMP-2), with human cells of a mesenchymal phenotype from two different origins: adipose tissue (hADSCs) and dental pulp (hDPSCs). The expression of osteogenic markers in 2D cultures was evaluated in several culture periods by means of the immunofluorescence technique and real-time gene expression analysis, taking as reference MG-63 cells of osteogenic origin. The same strategy was extrapolated to a 3D environment of polylactic acid (PLA), with a 3% alginate hydrogel. The expression of osteogenic markers was detected in both hADSCs and hDPSCs, cultured in either 2D or 3D environments. However, the osteogenic differentiation of MSCs was obtained based on the culture medium and the cell origin used, since higher osteogenic marker levels were found when hADSCs were cultured with medium supplemented with BMP-2. Furthermore, the 3D culture used was suitable for cell survival and osteogenic induction.

## 1. Introduction

Bone tissue provides support and protection to the different organs of our body [1, 2]. It is one of the most dynamic tissues of our organism and it is in constant remodeling, allowing not only adaptation to mechanical forces but also the repair of injuries. Under physiological conditions, bone has a great regenerative capacity, although this capacity is limited to small defects under specific conditions [3]. Among these adverse conditions for a successful repair, age-related osteoporosis becomes especially relevant, since at least 50% of the population suffers from chronic weakness and a deterioration in the quality of life due to osteoporosis, which also induces the appearance of microfractures [4]. Despite the great regenerative capacity of the bone, endogenous repair is not enough in some cases; thus, different therapeutical procedures have been developed [5]. The bone graft is one of the most widely used alternatives to repair bone fractures, since it allows a great regeneration in orthopedic procedures [6] and shows three essential biological properties for bone regeneration such as osteoconduction, osteoinduction, and osteogenesis [6]. In addition, an important criterion for evaluating the outcome of bone healing is osseointegration, which considers both the structural and functional bonds between the bone and the implant [7].

Conventional therapies show a low bone regeneration rate, chronic pain, and infection [8]. Hence, due to the few long-term successful results that these conventional therapies can produce, there is a growing interest in developing new therapeutic strategies that accelerate the physiological repair of fractures, such as tissue engineering techniques focused on treatment and repair of bone injuries [8, 9].

The main goal of tissue engineering is to combine living cells with 3D scaffolds and biologically active molecules to produce constructs that mimic the extracellular matrix and promote tissue repair and regeneration [10–12]. Therefore, the first critical step is the choice of a cell type. For bone regeneration, osteoblasts would be a good choice, but they are postmitotic cells *in vivo*, and *in vitro*, they show a low rate of proliferation and rapid programmed cell death [13]. On the other hand, mesenchymal stem cells (MSC) are an excellent alternative because they are capable of self-replicating and differentiating into specific cell lines such as bone cells, overcoming the disadvantages of using differentiated cells [14, 15], and indeed these pluripotent cells are widely used in regenerative medicine [16–18]. Bone marrow stem cells (BM-MSC) would be a suitable candidate for osteogenic regeneration, but the invasive procedure required to obtain them represents a significant disadvantage [19, 20]. For this reason, other cells with a mesenchymal phenotype, such as those located in adipose tissue (ADSCs) or dental pulp (DPSCs), represent an acceptable alternative [17, 21, 22]. Furthermore, DPSCs have shown greater potential in terms of proliferative and differentiation capacity than ADSCs [21].

The second step to take into account is the scaffolds and biomimetic materials used in bone tissue engineering [23] that are 3D structures that must provide the proper architecture and environment for the development and growth of bone tissue, guiding the complex process of fracture repair. The scaffolds are designed to promote cell proliferation, survival, adhesion, and migration, as well as to accelerate bone remodeling, provide an osteoconductive structural guide, and, in some cases, act as a carrier material for growth factors or antibiotics [11]. In fact, changes in their microstructure influence the response of MSCs and can modify, for example, cell adhesion to scaffold surfaces, cell proliferation, and osteogenic differentiation [23]. For these reasons, scaffolds must be biocompatible and noncytotoxic and with mechanical and surface properties similar to those of bone tissue [11, 24, 25]. Nowadays, both natural and synthetic polymers are used to produce these scaffolds. Natural polymers, such as alginate and hyaluronic acid, are widely used due to their biocompatibility and induction of cell growth [26, 27]; however, they lack the mechanical properties of bone [28]. Regarding synthetic polymers, poly(lactic) acid (PLA), poly(lactic-co-glycolic) acid (PLGA), and polycaprolactone (PCL) present the possibility of adjusting and modifying their mechanical properties [29]; however, they present drawbacks related to the lack of bioactivity, which restricts interactions with host tissue. A possible solution would be the manufacture of hybrid materials that improve cell adhesion, mineralization, and osteogenic differentiation [29]. However, this aspect has been poorly studied, and therefore, it is necessary to standardize the design and manufacturing methods of these hybrid materials to study the biological behavior of different stem cells cultured in them. In this regard, the use of commercially available 3D printing devices and materials could be of interest to rapidly generate prototypes suitable for *in vivo* research [30].

The final step includes growth factors, which are essential in the formation, maintenance, and regeneration of bone tissue, as they induce progenitor and inflammatory cells to migrate to the injury site and initiate the healing process [12, 26, 31]. Furthermore, these molecules increase scar formation, limit excessive bone formation, and accelerate the healing process [32]. For instance, platelet-rich plasma (PRP), which is enriched in natural and synthetic biomaterials, can be used in orthopedics in combination with autologous bone due to its effectiveness in wound healing processes [33]. Thus, osteogenic differentiation is based on the action of different growth factors such as PDGF, TGF*β*, FGF, IGF, and BMP, among others [30, 34]. BMP comprises a family of proteins of which BMP7 or BMP2 is of great importance in the control of bone formation [35, 36]. BMP2 plays a key role in the expression of osteogenic markers such as alkaline phosphatase (ALP) and osteocalcin (OC) [37, 38]. Furthermore, dexamethasone is known to induce MSC proliferation *in vitro* and their differentiation in the osteogenic lineage [39], although it induces less mineralization. For this reason, dexamethasone is used along with *β*-glycerophosphate and ascorbic acid [40]. While the former plays an important role in the mineralization process and in the modulation of osteogenic activities, ascorbic acid is essential to increase cell viability and stimulates osteogenic cells to produce type I collagen [11, 40].

Our aim in the present work was to study the inductive role of BMP2 in the osteogenic differentiation of human ADSCs and DPSCs (hADSCs and hDPSCs). The osteogenic potential of both cell types was studied in 2D cultures, analysing the expression of proteins related to osteogenesis (alkaline phosphatase and osteocalcin), calcium deposits in cell cultures, and cell morphology by means of the organization of the actin filament of the cytoskeleton. A comprehensive study of the relative levels of expression of different genes related to osteogenesis was also carried out. Finally, the bone differentiation potential of these cells *in vitro* was studied in a 3D environment. For this purpose, a 3D-printed scaffold prototype was manufactured with PLA and alginate, seeded with cells, and the bone-inductive capacity of BMP2.

## 2. Materials and Methods

### 2.1. Experimental Design

hADSC, hDPSC, and MG-63 cells were cultured in 2D environment with proliferation and osteogenic differentiation media (supplemented with BMP2 or with MesenCult reference induction medium) at a density of 2 × 10^3^ cells/ml for 2, 3, and 4 weeks. Alkaline phosphatase (ALP) and osteocalcin (OC) marker proteins were detected by immunofluorescence, and the cytoskeleton organization was studied by staining the actin filaments with rhodamine-conjugated phalloidin. Calcium deposits were analysed by staining with alizarin red. The relative expression of genes related to osteogenesis was analysed by real-time RT-PCR. Once the osteogenic model was evaluated in the 2D cultures, 8 × 10^6^ cells/ml were cultured for up to 3 weeks in scaffold prototypes consisting of a 3D-printed PLA framework embedded with alginate. In these scaffolds, the expression of osteocalcin measured by immunofluorescence was used as a marker of osteogenic differentiation.

### 2.2. Cell Culture

Human adipose-derived stem cells (hADSCs) and human dental pulp stem cells (hDPSCs) were purchased from Lonza (Basel, Switzerland), and MG-63 cells were from the American Type Culture Collection (ATCC® CRL-1427™; Sigma-Aldrich; Madrid, Spain). MG-63 cells are a cell line derived from osteosarcoma, which was chosen as a positive control for osteogenic differentiation [41]. The cells were cultured in 75 cm [2] flasks with proliferation medium composed of *α*MEM (Gibco; WA, USA) supplemented with 10% fetal bovine serum (FBS) (Gibco; USA), 1% penicillin-streptomycin (P/S) antibiotic solution (Gibco; USA), and 2 mM L-glutamine (Gibco; USA) for hADSCs and hDPSCs, while for MG-63 cells, this medium was supplemented with a 1% solution of nonessential amino acids (NEAA) (Gibco; USA) and 1% 100 nM sodium pyruvate (Gibco; USA). Osteogenic differentiation was induced by replacing the proliferation medium with an osteogenic differentiation media. Two different osteogenic differentiation media were used: the commercial osteogenic differentiation medium MesenCult™ (#05465 Stemcell Technologies; Grenoble, France) and a medium composed of *α*MEM supplemented with 10% FBS, 1% P/S antibiotic solution, 2 mM L-glutamine, 10 *μ*g/ml glycerol-2-phosphate (Merck; Darmstadt, Germany), 4 *μ*g/ml ascorbic acid (Merck; Germany), 0.1 *μ*g/ml dexamethasone (Merck; Germany), and 50 ng/ml of BMP2 (Stemcell Technologies; France). Cell cultures were maintained in a humidified atmosphere at 37°C, 5% CO_2_, and 95% air, replacing the culture media every 2-3 days.

### 2.3. Immunofluorescence

Cells were cultured on 8-well Millicell EZ slides (Merck) for 3 days in proliferation culture medium, and then, the medium was replaced with either osteogenic differentiation media (BMP2-supplemented or commercial MesenCult media) for up to 4 weeks. Samples were fixed with 4% paraformaldehyde in phosphate-buffered saline (PBS), pH 7.4, for 10 min at RT. After washing three times with PBS for 5 min at RT, cells were permeabilized with 0.1% Triton X-100 in PBS for 5 min and washed three times.

To detect the expression of ALP and OC proteins, the slides were incubated for 30 min with blocking solution (1% bovine serum albumin [BSA] and 1.1% Tween-20 in PBS). Then, they were incubated overnight at 4°C with mouse anti-human ALP or anti-human OC IgG antibodies (MAB 1448 and MAB 1419, respectively; R&D Systems; Minneapolis, USA) at 1 : 200 dilution in antibody diluent. The slides were then washed three times and incubated with FITC-conjugated rabbit anti-mouse IgG secondary antibody (D2883 Merck; Switzerland), diluted 1 : 200 in PBS, for 2 h, at RT in the dark, as previously reported [42].

To detect filamentous actin (F-actin), permeabilized slides were preincubated with PBS containing 1% BSA for 30 min to reduce nonspecific background. Then, a solution containing 200 *μ*l PBS and 5 *μ*l of rhodamine-conjugated methanolic phalloidin stock solution (Molecular Probes, Thermo Fisher Scientific; Waltham, MA, USA) was added to the samples and incubated for 20 min at RT, as previously reported [43].

Finally, the slides used for either ALC, OC, or F-actin detection were washed three times with PBS, and nuclear DNA was stained with 4′,6-diamidino-2-phenylindole (DAPI) (Sigma), and the samples were analysed using a Leica Biosystems 4000D fluorescence DM microscope (Leica Biosystems; Wetzlar, Germany) and photographed with the Leica DFC 340FX camera (Leica Biosystems).

### 2.4. Alizarin Red Staining

Calcium deposits were analysed by alizarin red staining as previously reported [44, 45]. Briefly, hADSC, hDPSC, and MG-63 cells were cultured on 8-well Millicell EZ slides (Merck) with osteogenic differentiation media (either BMP2-supplemented or commercial MesenCult media). After 4 weeks of culture, the cells were fixed with 4% paraformaldehyde PBS, pH 7.4, for 10 min at RT, washed twice with distilled water, and covered with alizarin red solution (2% alizarin S in distilled water, pH 4.2) for up to 15 min at RT. The samples were washed twice with distilled water and stained with hematoxylin for 10 s. Finally, the cells were washed twice, and a standard dehydration with ethanol solutions (50-70-100%) and two final steps in xylol was performed. The samples were mounted and studied using a Leica DMBL optical microscope (Leica Biosystems; Germany). Pictures were captured with a Leica ICC50 digital camera (Leica Biosystems).

### 2.5. Real-Time RT-PCR

The expression of specific osteogenic genes was studied by real-time RT-PCR. RNA was isolated and purified, and reverse transcription (RT) was carried out using the high-capacity cDNA synthesis kit (Life Technologies; Madrid, Spain), following the manufacturer's instructions.

The cDNA was amplified by real-time PCR using the Universal Gene Expression Master Mix (Life Technologies; Spain) and a 7900HT real-time Thermocycler (Applied Biosystems; CA, USA) according to the supplier's instructions. For amplifications, the following predesigned Assays on Demand (Life Technologies; Spain) were used: BGLAP (Hs01587813_g1), CSF1 (Hs00174164_m1), CSF2 (Hs00929873_m1), IBSP (Hs00913377_m1), MT-CO1 (Hs02596864_g1), RUNX2 (Hs01047973_m1), and SPP1 (Hs00959010_m1). GAPDH (Hs02786624_g1) was used as a housekeeping gene. The relative expression of each gene was calculated using the comparative *ΔΔ*C_T_ method. All reactions were performed in triplicates.

### 2.6. PLA Scaffold Manufacture, Characterization, and Sanitization

For 3D printing, porous scaffolds were designed using Tinkercad™ application (Autodesk; California, USA) with the Cura 2 software (Ultimaker Cura Software). Different manufacturing parameters were considered, such as number of walls [2] ,thickness of the walls (1 mm), thickness of the bottom and the top (0 mm), density of the filling (45%), filling pattern (lines), and printing speed (60 mm/s). The scaffolds were made using the BQ Hephestos 2 printer (BQ; Madrid, Spain) with a 0.4 mm nozzle extruder coupled to a PLA coil (1.75 mm diameter filament).

For the characterization of the scaffold, images were taken in a JSM-5410 scanning electron microscope (Jeol; Tokyo, Japan) at 15 mm distance and 15 kV voltage. Five images of 3 scaffolds were processed to measure mean pore size with the ImageJ/FIJI software, using the Nearest Neighbor Distance plug-in. Three samples of PLA and several samples with different thicknesses were used for the tensile test. Three specimens of 1.5 mm and 2.6 mm and four of 3.1 mm specimens were used. All of them were 15.5 mm wide, and the jaw part was 60 mm long. The Dy34 traction equipment (Adamel Homargy Division D'instruments SA; Ivry, France) was used with a 1 kN load cell. An initial speed of 10 mm/min was applied, and the data were obtained using TestWorks 4® software (MTS Systems; Minnesota, USA).

After those studies, the scaffolds were sanitized by sequential washings with 100-100-70-50-30% ethanol (previously filtered with a 0.2 *μ*m pore filter) and sterile ultrapure water, for 1 h each under constant 390 rpm agitation. Then, the samples were washed with PBS twice for 1 h each. Finally, the scaffolds were dried inside the cell incubator.

### 2.7. Cytotoxicity Assay of 3D-Printed Scaffolds

Conditioned medium was obtained by incubating 3D-printed scaffolds with proliferation medium without phenol red, at 37°C and shaking at 1,500 rpm for 24 h and 7 days. Cytotoxicity was tested by incubating MG-63 cells with conditioned medium, as previously reported [46, 47]. Briefly, MG-63 cells were seeded in a 96-well plate at a density of 1 × 10^4^ cells/well. After 24 h, the culture medium was replaced with scaffold-conditioned medium. Latex-conditioned medium was used as a positive control of cytotoxicity. The cells were then cultured for an additional 24 h. Then, the MTS assay (Sigma-Aldrich; Madrid, Spain) was carried out by adding 20 *μ*l of MTS reagent to each well and incubating for 2 h. Finally, absorbance was measured at 490 nm on a Victor X3 2.030 multilabel reader (PerkinElmer; Massachusetts, USA). The absorbance value is directly proportional to cell viability.

### 2.8. Manufacture of Mixed Hydrogel-PLA Scaffold-Containing Cells

The 3D-printed PLA scaffolds were embedded with cells suspended in alginate as follows. A 3% alginate solution was prepared with ultrapure water containing 40 mM HEPES and 300 mM NaCl, pH 7.4, and sterilized at 121°C and 1 atm for 20 min. Then, hADSCs or hDPSCs (8 × 10^6^ cells/ml) were added to the alginate solution prewarmed to 37°C. Scaffolds were placed in 12-well culture plates and embedded with 100 *μ*l of the alginate solution containing the cell suspension. Then, the polymerization of alginate was induced by adding a solution containing 102 mM CaCl_2_ and 10 mM HEPES in sterile distilled water at a concentration of 10% with respect to the final volume of alginate per sample. The scaffold-containing cells were incubated for 30 min at RT and then immersed in proliferation medium. After 3 days of culture, the medium was replaced with osteogenic differentiation media (BMP2-supplemented or commercial MesenCult), and the seeded scaffolds were cultured for up to 3 additional weeks. The culture medium was replaced every 2-3 days.

### 2.9. Data Presentation

All experiments were done in triplicate. The figures presented in the manuscript are representative of the results. Relative expression data was analysed using GraphPad Prism 5.0 software (GraphPad Software Inc.). The analysis of the parametric test of variance (ANOVA) was carried out. The differences were considered statistically significant for values of *p* ≤ 0.05.

## 3. Results

### 3.1. BMP2-Supplemented Medium Induces the Reorganization of Cytoskeleton and the Expression of the ALP and OC Proteins

**Figure 1:**
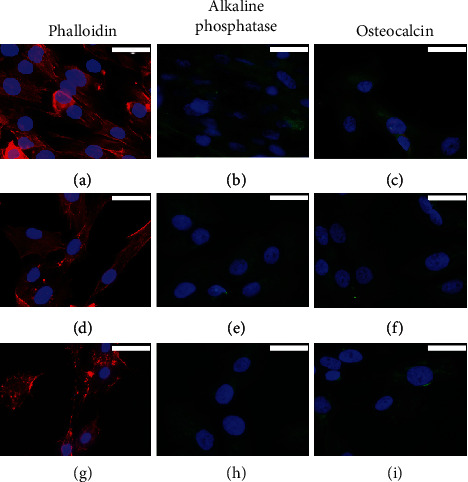
Rhodamine-phalloidin staining and immunofluorescence of alkaline phosphatase (ALP) and osteocalcin (OC) in 2D-cultured MG-63 cells for 4 weeks with (a–c) proliferation medium or with (d–f) commercial MesenCult or (g–i) BMP2-supplemented osteogenic differentiation media. Cell nuclei were stained with DAPI and observed in blue, the distribution of actin filaments in red, and the presence of ALK or OC in green. Scale bar = 25 *μ*m.

**Figure 2:**
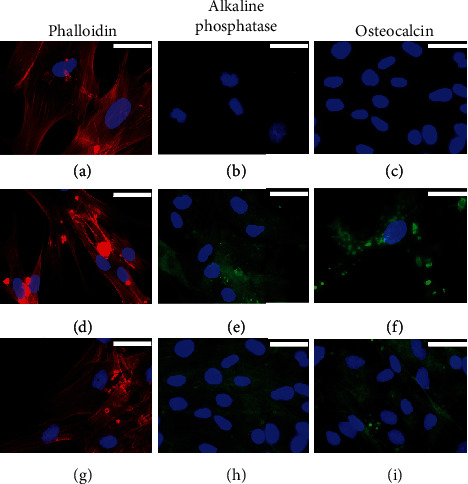
Rhodamine-phalloidin staining and immunofluorescence of alkaline phosphatase (ALP) and osteocalcin (OC) in 2D-cultured hADSCs for 4 weeks with (a–c) proliferation medium or with (d–f) commercial MesenCult or (g–i) BMP2-supplemented osteogenic differentiation media. Cell nuclei were stained with DAPI and observed in blue, the distribution of actin filaments in red, and the presence of ALK or OC in green. Scale bar = 25 *μ*m.

**Figure 3:**
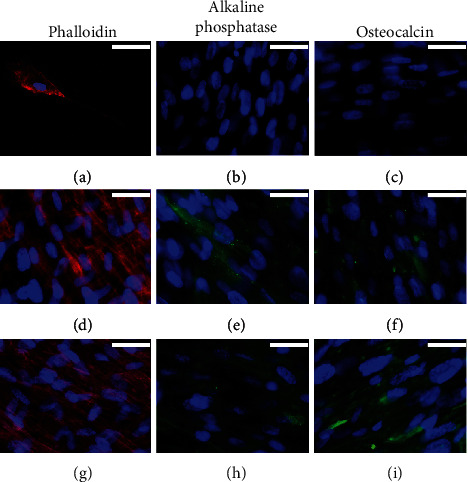
Rhodamine-phalloidin staining and immunofluorescence of alkaline phosphatase (ALP) and osteocalcin (OC) in 2D-cultured hDPSCs for 4 weeks with (a–c) proliferation medium or with (d–f) commercial MesenCult or (g–i) BMP2-supplemented osteogenic differentiation media. Cell nuclei were stained with DAPI and observed in blue, the distribution of actin filaments in red, and the presence of ALK or OC in green. Scale bar = 25 *μ*m.

We studied the osteogenic induction capacity of BMP2-supplemented culture medium in both types of mesenchymal cells, hADSC and hDPSC, in 2D cultures for up to 4 weeks. The MG-63 cell line was included in the study as control of cells of osteogenic lineage. Commercial MesenCult medium was used as a reference inducer for the *in vitro* differentiation of human mesenchymal stem cells into cells of osteogenic lineage. The results obtained are summarized in Figures 1–3.

Cytoskeletal analysis in MG-63 cells revealed discrete changes in the three experimental groups analysed (proliferation medium and osteogenic differentiation BMP2-supplemented or commercial MesenCult media). In cells cultured with proliferation medium, actin was observed to form a 3D network of long filament through the cytoplasm, which accumulated in bundles on the periphery of the cells just below the plasma membrane, but when osteogenic differentiation medium was added, F-actin staining decreased in the cell cytoplasm while an increase in stress fibers was observed at the cell periphery, with the appearance of cellular contacts as indicated by the focal accumulation of stained actin fibers. These changes were more evident in cells cultured with BMP2-supplemented medium compared to the commercial MesenCult culture medium (Figures 1(a), 1(d), and 1(g)).

When MG-63 cells were cultured with proliferation culture medium, a low expression of ALP and OC was shown in the cytoplasm. However, a significant increase in the expression of ALP and OC proteins was observed in MG-63 cells cultured with both osteogenic differentiation media, the commercial MesenCult, and the BMP2-supplemented media. For ALP, no differences were observed between both osteogenic media (Figures 1(b), 1(e), and 1(h)). With respect to OC, only a slight increase was observed when the cells were cultured with the medium supplemented with BMP2 (Figures 1(c), 1(f), and 1(i)).

A similar trend was observed for hADSCs. In cells cultured with proliferation medium, actin filaments were observed crossing the cytoplasm, with bundles below the plasma membrane. Both osteogenic differentiation media induced the formation of peripheral stress fibers and a greater presence of intercellular contacts (Figures 2(a), 2(d), and 2(g)). The expression of the ALP protein was not detected in cells cultured in proliferation medium (Figure 2(b)). Although both osteogenic differentiation culture media stimulated the expression of this protein, the commercial MesenCult medium appeared to be more efficient than BMP2-supplemented medium (Figures 2(e) and 2(h)). A marked increase in the expression of OC protein was also observed when cells were cultured in both osteogenic differentiation media studied, compared to cells cultured in proliferation medium, in which the expression of this protein was not detected (Figures 2(c), 2(f), and 2(i)).

For hDPSC culture, the changes in the cytoskeleton were difficult to evaluate due to the high proliferation of these cells, and thus, it was difficult to establish differences between the experimental groups (Figures 3(a), 3(d), and 3(g)). The expression of both ALP and OC proteins was not detected in cells cultured with proliferation medium; however, both osteogenic induction media significantly increased the expression of ALP and OC in a similar way (Figures 3(b), 3(c), 3(e), 3(f), 3(h), and 3(i)).

### 3.2. BMP2-Supplemented Medium Induces Calcium Deposition in Cell Cultures

**Figure 4:**
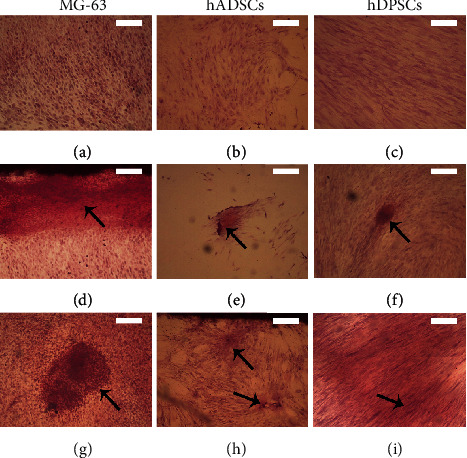
Alizarin red staining of MG-63 cells, hADSCs and hDPSCs cultured in 2D for 4 weeks with (a–c) proliferation medium or with (d–f) commercial MesenCult or (g–i) BMP2-supplemented osteogenic differentiation media. Cell nuclei were stained with hematoxylin. Calcium deposits are showed in red (arrows). Scale bar = 200 *μ*m.

Calcium deposition is a relevant indicator of the activity of bone-forming cells. To the study of calcium deposits, alizarin red staining was used in MG-63 cells, hADSC and hDPSC 2D-cultured with proliferation medium or with commercial MesenCult or BMP2-supplemented osteogenic differentiation media, for up to 4 weeks. No calcium deposits were observed in any of the cell types studied when cultured with proliferation medium (Figures 4(a)–4(c)). However, both MesenCult and BMP2-supplemented osteogenic differentiation media strongly induced the deposition of calcium in MG-63 cells as well as in both types of stem cells studied, although it was higher in hDPSCs compared to hADSCs (Figures 4(d)–4(i)).

### 3.3. BMP2-Supplemented Medium Induces the Gene Expression of Bone-Related Markers

**Figure 5:**
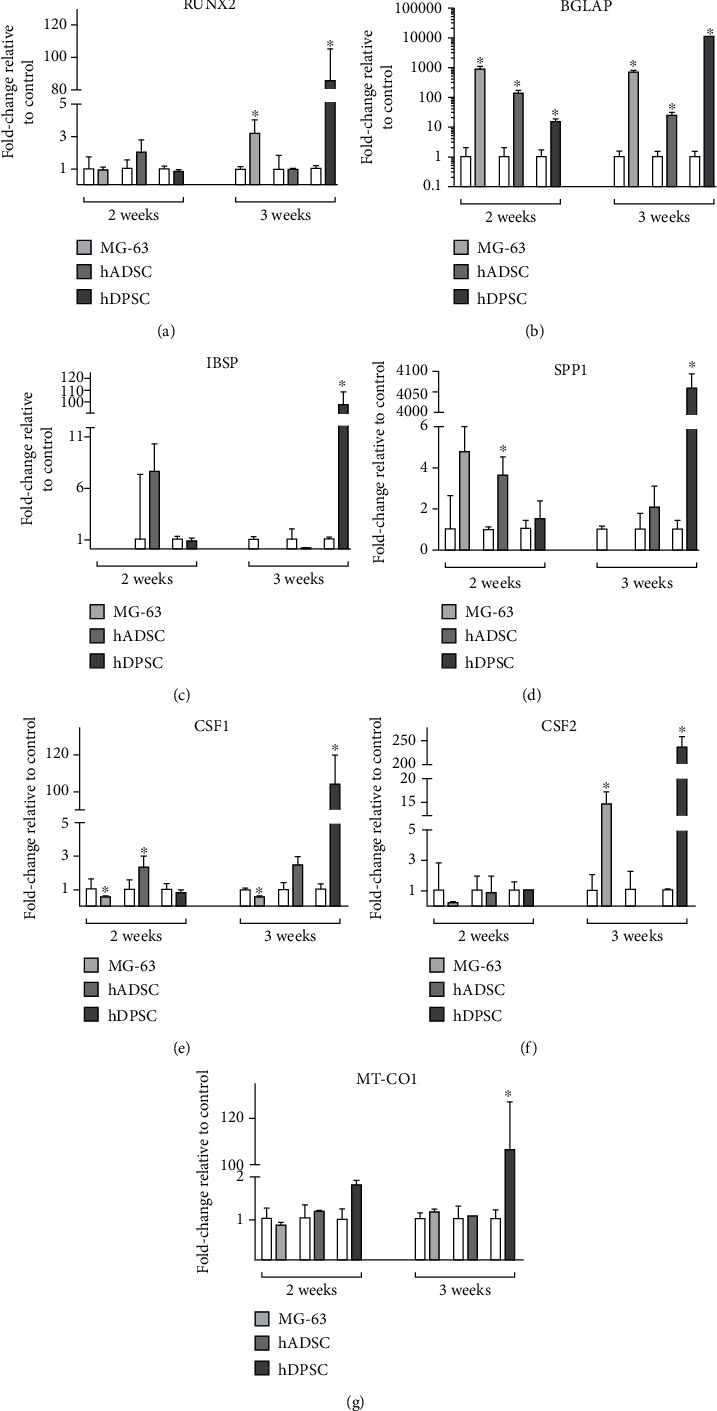
Relative levels of gene expression of 2D-cultured MG-63 cells, hADSCs and hDPSCs, for 2 and 3 weeks with proliferation medium or commercial MesenCult osteogenic differentiation medium of (a) RUNX2, (b) BGLAP, (c) IBSP, (d) SPP1, (e) CSF1, (f) CSF2, and (g) MT-CO1. Samples cultured with proliferation media were taken as relative control. Mean ± SD are shown, with statistical significance at ^∗^*p* ≤ 0.05 with respect to the control group.

**Figure 6:**
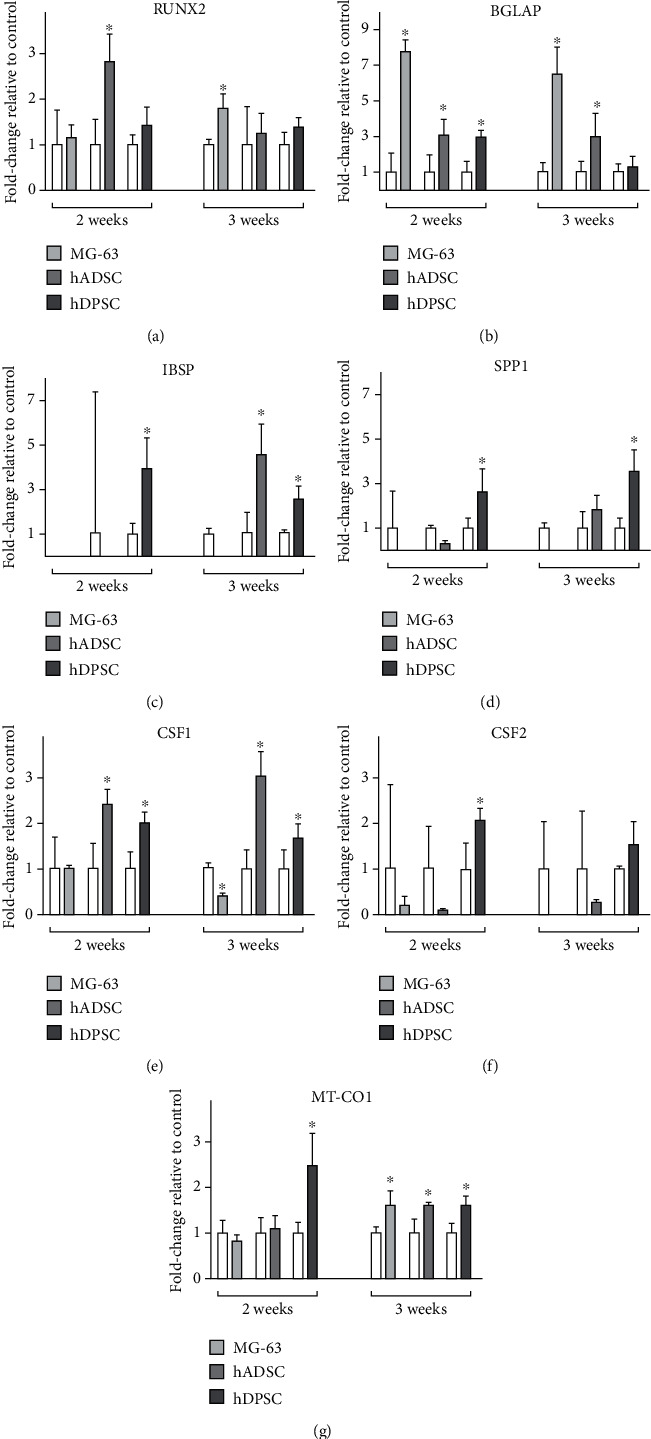
Relative levels of gene expression of 2D-cultured MG-63 cells, hADSCs and hDPSCs, for 2 and 3 weeks with proliferation medium or BMP2-supplemented osteogenic differentiation medium of (a) RUNX2, (b) BGLAP, (c) IBSP, (d) SPP1, (e) CSF1, (f) CSF2, and (g) MT-CO1. Samples cultured with proliferation media were taken as relative control. Mean ± SD are shown, with statistical significance at ^∗^*p* ≤ 0.05 with respect to the control group.

The expression of RUNX2 (Figures 5(a) and 6(a)) increased in MG-63 cells when cultured for 3 weeks with both osteogenic differentiation media, showing a significant 3-fold increase when cultured with commercial MesenCult medium and 2-fold when cultured with BMP2-supplemented medium, when compared with cells cultured with proliferation medium. The expression of RUNX2 in hADSCs was significantly higher when cultured with BMP2-supplemented osteogenic medium for 2 weeks, showing a value close to 3-fold increase with respect to the control samples cultured with proliferation medium; however, no differences were observed when cultured with commercial MesenCult osteogenic differentiation medium. In the case of hDPSCs, a significant 90-fold increase was observed when cultured with commercial MesenCult medium for 3 weeks compared to the control, but no differences were observed when cultured with BMP2-supplemented medium at any time studied.

In the case of BGLAP gene expression (Figures 5(b) and 6(b)), high increases in expression were observed at both times of culture studied, with both osteogenic differentiation media analysed. For MG-63 cells cultured for 2 and 3 weeks with MesenCult medium, gene expression increases almost 900-fold, while hADSCs showed an increase close to 100-fold after 2 weeks of culture with MesenCult medium and to 30-fold after 3 weeks. Finally, hDPSCs cultured with MesenCult medium showed an increment close to 20-fold after 2 weeks of culture and a large increase of 10,000-fold when cells were cultured for 3 weeks. When cells were cultured with BMP2-supplemented medium for 2 weeks, BGLAP expression showed a significant 8-fold increase for MG-63 cells and 3-fold increase for both hADSCs and hDPSCs. These high levels of expression remained significantly increased after 3 weeks of culture for MG-63 cells and hADSCs.

The expression of IBSP marker gene (Figures 5(c) and 6(c)) increased in hDPSCs cultured for 2 weeks with BMP2-supplemented osteogenic differentiation medium, showing a significant 4-fold increase with respect to the controls, cultured with proliferation medium. After 3 weeks of culture with osteogenic differentiation media, these cells showed a large increase close to 100-fold when cultured with MesenCult medium and to 3-fold with BMP2-supplemented medium. However, hADSCs only showed a significant increase in the expression of this gene when cultured for 3 weeks with BMP2-supplemented medium, showing a 5-fold increase compared to controls.

For the expression of SPP1 (Figures 5(d) and 6(d)), hADSCs showed a 4-fold increase when cultured with MesenCult medium for 2 weeks. In the case of hDPSC culture, they showed a huge, significant 4,000-fold increase when cultured for 3 weeks with MesenCult medium and milder 3- and 4-fold increases for 2 and 3 weeks of culture, respectively, when cultured with BMP2-supplemented osteogenic differentiation medium.

In the case of CSF1 gene expression (Figures 5(e) and 6(e)), hADSCs and hDPSCs cultured with BMP2-supplemented osteogenic differentiation medium showed a significant increase close to 3-fold change in the samples cultured for 2 and 3 weeks. However, when hDPSCs were cultured with commercial MesenCult medium, a significant increase close to 100-fold was observed only at 3 weeks of study, while hADSCs showed a slight but significant increase only at 2 weeks of culture. On the other hand, MG-63 cells cultured in both osteogenic differentiation media for 3 weeks showed significant decreases of CSF1 expression.

Regarding CSF2 expression (Figures 5(f) and 6(f)), hDPSCs cultured with the commercial MesenCult medium for 3 weeks showed a significant increase of over 200-fold with respect to controls, while the increase was milder when cultured with BMP2-supplemented medium for 2 weeks, showing a relative increase of CSF2 expression close to 2-fold the control values. MG-63 cells showed a 15-fold increase in expression values when cultured for 3 weeks with commercial MesenCult medium, but no significant differences in CSF2 expression were observed with other cells or media used.

Finally, MT-CO1 marker gene expression (Figures 5(g) and 6(g)) showed a significant increase close to a 3-fold value in hDPSCs cultured for 2 weeks with BMP2-supplemented osteogenic differentiation medium, while after 3 weeks with this culture medium, all cell types showed a significant 2-fold increase. However, in cells cultured with commercial MesenCult osteogenic differentiation medium, MT-CO1 expression only varied in hDPSCs cultured for 3 weeks, with a high increase close to 100-fold compared to controls.

### 3.4. Manufacture and Characterization of 3D-Printed PLA Scaffolds

A prototype consisting of a 3D-printed PLA scaffold was manufactured in order to study the osteogenic induction ability of BMP2 in 3D environments. The Tinkercad software was used to design scaffolds consisting of 10 × 10 × 3 mm cubes for cell cultures or 60 × 15.5 × 1.5 − 3 mm cubes for mechanical characterization studies. STL files were generated, and lamination was carried out using Cura 2 software according to the parameters described in Materials and Methods, and the prototypes were printed using a Hephestos 2 printer.

The biomechanical characterization of the scaffolds is summarized in Table 1. Three different scaffolds were printed, and average ± SD is represented.

**Figure 7:**
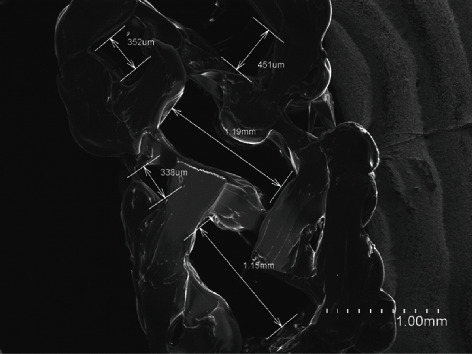
Ultrastructural organization of the printed scaffolds. Images were taken in a JSM-5410 scanning electron microscopy (SEM). Scale bar = 1 mm.

The ultrastructural organization of the printed scaffolds was studied using scanning electron microscopy. A representative image is shown in Figure 7. The obtained scaffolds had a porosity of 55%, with an average pore size of 1.1 mm in length and a density of pores of 1 pore/mm [2].

Finally, regarding the cell toxicity, MG-63 cells were cultured with conditioned culture medium as described in Materials and Methods, for up to 72 h. The cytotoxicity assay did not show significant differences between cells cultured with nonconditioned medium and with conditioned medium. However, when cells were cultured with latex-conditioned medium, used as a positive control for cytotoxicity, cell viability revealed a significant 95% decrease of living cells (data not shown).

### 3.5. BMP2 Induces Osteogenic Differentiation in Mixed 3D-Printed PLA-Alginate Scaffolds

Finally, we studied the osteogenic differentiation ability of BMP2 in a mixed 3D-printed alginate scaffold. MG-63 cells, hADSCs and hDPSCs, were cultured in 3D scaffolds as described above. The scaffold-containing cells were cultured for up to 3 weeks with different media: proliferation medium or osteogenic differentiation media (commercial MesenCult or BMP2-supplemented). The organization of the cytoskeleton was studied by fluorescence staining of F-actin with rhodamine-phalloidin. Osteocalcin was selected as a marker of osteogenic differentiation and detected by immunofluorescence.

**Figure 8:**
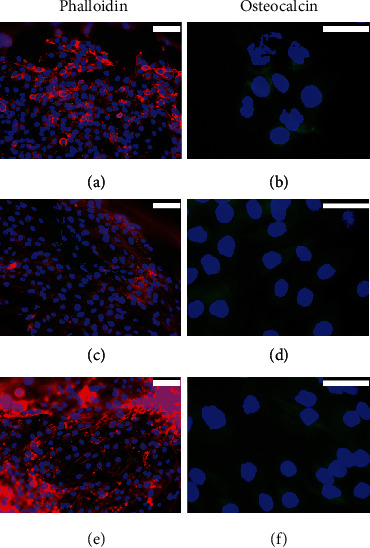
Rhodamine-phalloidin staining and OC immunofluorescence in 3D-cultured MG-63 cells for 3 weeks with (a, b) proliferation medium or with (c, d) commercial MesenCult or (e, f) BMP2-supplemented osteogenic differentiation media. Cell nuclei were stained with DAPI and observed in blue, the distribution of actin filaments in red, and the presence of OC in green. Scale bar = 75 (a, c, and e) and 25 *μ*m (b, d, and f).

Figure 8 shows MG-63 cells in the manufactured PLA scaffolds. It is observed that the cells occupied the pores of the scaffolds, and the number of cells was higher in the scaffolds cultured with both proliferation medium and BMP2-supplemented osteogenic differentiation medium, compared to those cultured with commercial MesenCult osteogenic differentiation medium (Figures 8(a), 8(c), and 8(e)). The presence of OC was detected in MG-63 cells cultured in the different media analysed, but no differences were observed regarding fluorescence intensity or distribution pattern between the groups studied (Figures 8(b), 8(d), and 8(f)).

**Figure 9:**
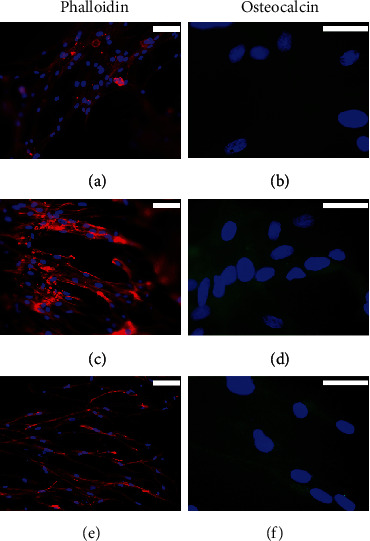
Rhodamine-phalloidin staining and OC immunofluorescence in 3D-cultured hADSCs for 3 weeks with (a, b) proliferation medium or with (c, d) commercial MesenCult or (e, f) BMP2-supplemented osteogenic differentiation media. Cell nuclei were stained with DAPI and observed in blue, the distribution of actin filaments in red, and the presence of OC in green. Scale bar = 75 (a, c, and e) and 25 *μ*m (b, d, and f).

When hADSCs were cultured in PLA scaffolds, no differences were observed in the number of cells between the different media used (Figures 9(a), 9(c), and 9(e)); however, the cell density was lower than that observed with MG-63 cells. Unlike MG-63 cells, the presence of OC was not detected in hADSCs cultured with proliferation medium (Figure 9(b)); however, both osteogenic induction media induced a slight but similar expression of OC (Figures 9(c) and 9(e)).

**Figure 10:**
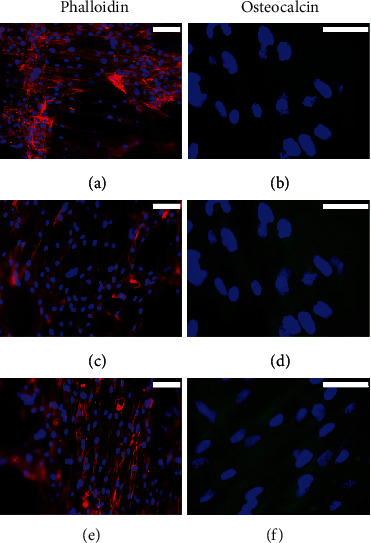
Rhodamine-phalloidin staining and OC immunofluorescence in 3D-cultured hDPSCs for 3 weeks with (a, b) proliferation medium or with (c, d) commercial MesenCult or (e, f) BMP2-supplemented osteogenic differentiation media. Cell nuclei were stained with DAPI and observed in blue, the distribution of actin filaments in red, and the presence of OC in green. Scale bar = 75 (a, c, and e) and 25 *μ*m (b, d, and f).

With respect to the hDPSCs, they showed higher number of cells after 3 weeks of culture than the hADSCs in all the culture media used (Figures 10(a), 10(c), and 10(e)). OC expression was like that of hADSCs: when cells were cultured with proliferation medium, no OC expression was observed, while this protein was present when cells were cultured with both osteogenic differentiation media (Figures 10(b), 10(d), and 10(f)).

## 4. Discussion

The human skeleton is made up mainly of bone tissue, which supports and protects different organs and tissues [1, 2]. It loses effectiveness during aging, and some pathologies can also increase its fragility and cause fractures [48]. Although bone has the capacity for continuous renewal throughout life, the ideal conditions for spontaneous healing do not always exist [49]. Currently, bone grafts are being used as a solution to these injuries, which promote bone healing through a variety of osteoconductive, osteoinductive, and osteogenic mechanisms, but they have several limitations.

In the present work, we have evaluated the optimal conditions for the *in vitro* differentiation of MSCs to osteogenic cells. For this reason, the effect of the osteogenic differentiation medium supplemented with dexamethasone, ascorbic acid, glycerol-2-phosphate, and BMP2 was studied in two types of mesenchymal cells, hADSCs and hDPSCs. Furthermore, we used MG-63 cells as a control and the commercial MesenCult osteogenic differentiation medium as a reference culture medium. These osteogenic differentiation culture media have been shown to induce the differentiation of MSCs in cells of the osteogenic lineage, and therefore, the main goal of this study was to evaluate and compare their osteogenic induction activity, and thus their role in fracture repair [50]. Once the efficacy of the inducing media was demonstrated, the same model was extrapolated to a 3D environment, for its future application as therapy for bone lesions.

After 4 weeks of cell culture, we studied the expression of two fundamental bone proteins, such as alkaline phosphatase (ALP) and osteocalcin (OC) [41, 51, 52], which play an important role during the formation and maturation of new bone. The commitment of MSCs to an osteogenic lineage depends on factors such as BMP and TGF*β*1, which regulate the expression of specific markers in the transition from preosteoblasts to osteoblasts (such as ALP) or mature osteoblast markers such as OC [53, 54]. On the one hand, we observed the expression of ALP and OC in MG-63 cells when they were cultured in both proliferation and osteogenic differentiation media. On the other hand, we observed that hADSCs and hDPSCs cultured in proliferation medium did not express neither of the two markers. However, their synthesis was detected when both cell lineages were cultured with osteogenic inductor media. Furthermore, it is important to point out that MSCs cultured with commercial MesenCult osteogenic differentiation medium showed a more intense ALP and OC synthesis than those cultured with BMP2-supplemented medium. These results agree with those previously reported by Ravichandran et al. [55] and Kraft et al. [56], who also detected the expression of both markers in MSCs cultured with osteogenic differentiation medium, thus demonstrating that osteogenic differentiation medium induces changes in the phenotype of MSCs associated with the osteogenic differentiation.

As Goncharenko et al. indicate that there are other characteristics such as cell shape, mechanical properties, or the organization of the cell cytoskeleton that play an important role in cell differentiation [57]. In our work, the morphology of the MSCs was examined by phalloidin staining to study possible changes promoted by the culture media. MG-63 cells and hADSCs did not show cell shape differences when cultured in the different media used. However, we observed differences in actin filaments in hADSC samples, where the filaments were thicker in cells cultured in both osteogenic differentiation media than in those cultured in proliferation medium. However, it was not possible to determine the cell morphology in phalloidin-stained hDPSCs since the high cell confluence did not allow us an adequate visualization. Thus, we can affirm that there is a tendency for the cultured cells to maintain a constant shape, although there seem to be changes in the thickness of the actin filaments, according to Titushkin and Cho, who described a reorganization of the cytoskeleton when the MSCs were exposed to an osteogenic stimulation [58] and reported that osteogenic differentiation media promoted the replacement of MSC stress fibers by thin actin fibrils, which form network-like structures that are characteristic of mature osteoblasts. These findings are compatible with the results presented here, since we observed a decrease in intracellular phalloidin staining and the formation of peripheral stress fibers, as well as an increase of focal cell contacts, which are characteristics of mature osteoblasts. Thus, if MSCs are induced osteogenically, changes in the dynamics of cytoskeletal reorganization occur, and chemical factors such as dexamethasone have been reported to be responsible [59].

Therefore, another osteogenic marker such as bone mineralization was evaluated [60]. L-type voltage-dependent Ca^2+^ channels (VDCCL) contribute to functional activities of osteoblasts and are also present in MSCs, playing an important role in osteogenic differentiation [61]. Thus, the presence of Ca^2+^ deposits in cells cultured for 4 weeks with osteogenic differentiation media was analysed by alizarin red S staining. MG-63 cells stained with alizarin red showed the presence of large Ca^2+^ deposits of an intense red color. The same staining was observed for hADSCs, but with smaller deposits than in MG-63 cells, indicating a lower degree of mineralization. In hDPSCs, large stained areas were observed but with low intensity, which indicates a moderate deposition of Ca^2+^, which does not agree with Ravichandran et al. [55], who reported large deposits. This could be due to our low number of cells seeded on the culture dish, associated with the proliferation capacity.

Then, the analysis of osteogenic gene expression was carried out by means of real-time RT-PCR. Cells cultured with osteogenic differentiation media were compared with those cultured with proliferation medium, which were taken as a control reference. MG-63 cells cultured with both osteogenic differentiation media for 2 and 3 weeks showed increased expression of BGLAP, which encodes osteocalcin, a specific marker protein for osteoblast maturation since it is synthesized exclusively by this cell type [62]. At 3 weeks, there was a slight increase in the expression of RUNX2 and MTCO1 in MG-63 cells. While RUNX2 participates in the commitment of MSC to osteogenic lineage [63], MTCO1 is associated with prostaglandin production under homeostatic conditions, which plays an important role in osteogenesis [64]. After 2 weeks of culture in osteogenic differentiation media, hADSCs showed expression of BGLAP, CSF1, and RUNX2. CSF1 is responsible for inducing osteoclastogenesis and inhibits the bone formation process of osteoblasts [65]. After 3 weeks of hADSC culture, BGLAP expression was maintained, with an increase in the expression of IBSP gene that encodes bone sialoprotein, playing an important role in bone mineralization [62]. Finally, hDPSCs cultured for 2 and 3 weeks with osteogenic differentiation media showed an increase in gene markers BGLAP, IBSP, and SPP1. SPP1 encodes osteopontin, an important protein for osteoblast maturation, since it participates in the transition from preosteoblast to osteoblast [66].

Briefly, the commercial MesenCult osteogenic differentiation medium promoted a higher increase in osteogenic gene markers such as RUNX2, BGLAP, and IBSP compared to proliferation medium or to BMP2-supplemented osteogenic differentiation medium. However, BMP2-supplemented medium showed higher values of increase in osteogenic differentiation after 3 weeks of culture. For that reason, although commercial medium improves early cell differentiation, both osteogenic culture media could be used for osteogenic differentiation of MSCs.

Once the osteogenic differentiation potential of hADSCs and hDPSCs was studied in the 2D model, these cells were cultured in a 3D environment to analyse their osteogenic potential in a mechanically and morphologically similar structure to bone. Initially, PLA was selected as a material to be used in the manufacture of scaffolds, due to its usability as a biodegradable polymer in tissue engineering [25]. Resorbable biomaterials such as PLA have many advantages for bone tissue engineering, including the ability to release substances such as drugs or growth factors intended to increase osteointegration of implanted materials [67]. In this sense, it would be possible to design a scaffold based on the one proposed in this work with the ability to release BMP2. For the above reasons, PLA was used to print scaffolds using a home 3D printer. The scaffold structure was designed taking care to porosity, with a value of 55%, which is similar to 50-90% of human bone porosity [10]. The characterization of the scaffolds showed that the percentage of porosity had very similar values regarding the percentage of tensile deformation. Additionally, the cytotoxicity assay guaranteed that this material is innocuous.

Cell culture with alginate hydrogel on PLA scaffolds improved the osteogenic differentiation capacity of the cells under study. The effectiveness of the alginate-based hydrogel was demonstrated in a large number of regeneration processes [42, 68, 69]. After 3 weeks of MG-63 cell culture, OC expression was detected in cells cultured in proliferation as well as in osteogenic differentiation media. However, both hADSCs and hDPSCs showed OC expression in both osteogenic differentiation media but not in proliferation medium, like that previously observed in 2D cultures. Other important observation is the increase in the number of MG-63 cells and hDPSCs that was observed in cultures in the 3D environment. Cell density is one of the key aspects that affect the regeneration of skeletal tissue, due to the importance of cell proliferation as well as differentiation in tissue engineering. In this regard, hDPSCs showed better results than hADSCs.

Therefore, the results of this study demonstrate the efficacy of hADSCs and hDPSCs in bone tissue regeneration therapies, due to their adequate proliferation and regeneration capacity. Furthermore, we present a new field of research because there are no previous studies to our knowledge that compare cell types and different osteogenic stimuli. This feature is of vital importance to find the most suitable culture conditions to achieve an optimal osteogenic differentiation state, thus ensuring the effectiveness in *in vivo* therapy.

## 5. Conclusions

Osteogenic differentiation *in vitro* has been successfully achieved in 2D cultures with the two media analysed, the first one from Stemcell Technologies and the second one supplemented with BMP2. This was corroborated by evaluating the expression of osteogenic markers with gene analysis techniques using real-time RT-PCR and immunostaining. We demonstrate that the composition of the culture medium directly affects the efficiency of the osteogenic differentiation process of MSCs, the MesenCult being the most suitable culture medium for hADSCs and BMP2-supplemented medium for hDPSCs. Significant changes have been shown between both types of cells, incubated in the same culture medium under the same conditions.

Finally, and according to the literature, 3D models improve survival and osteogenic induction of MSCs. However, further experimental trials with 2D cultures will be necessary before 3D, to guarantee the effectiveness of this process. Subsequently, it will also be important to consider the suitability of some materials used in the manufacture of 3D constructs, as well as a good choice of the animal model for a possible *in vivo* application.

## Figures and Tables

**Table 1:** Values of the mechanical properties of PLA scaffolds.

	60 × 15.5 × 1.5 mm	60 × 15.5 × 2.5 mm	60 × 15.5 × 3.0 mm
Fracture tensile stress (MPa)	6.8 ± 0.2	9.0 ± 0.3	10.2 ± 0.2
Ultimate stress (%)	3.8 ± 0.3	4.3 ± 0.3	3.7 ± 0.3
Maximum load at breaking point (N)	158.8 ± 4.7	364.2 ± 13.9	489.2 ± 7.2
Young's modulus (MPa)	2.6 ± 0.1	3.2 ± 0.1	3.6 ± 0.2
Tensile strength (MPa)	6.9 ± 0.2	9.2 ± 0.2	10.3 ± 0.1
Maximum stress strain (%)	3.5 ± 0.1	4.0 ± 0.5	3.5 ± 0.2
Elastic limit (MPa)	6.5 ± 0.1	8.7 ± 0.4	10.1 ± 0.2
Yield stress (%)	2.5 ± 0.1	2.7 ± 0.1	2.8 ± 0.2

## Data Availability

The data used to support the findings of this study are included in the article.
